# *Leucocalocybe mongolica* Fungus Enhances Rice Growth by Reshaping Root Metabolism, and Hormone-Associated Pathways

**DOI:** 10.1186/s12284-025-00813-4

**Published:** 2025-06-16

**Authors:** Mingzheng Duan, Ming Tao, Fuhan Wei, Honggao Liu, Sirui Han, Jieming Feng, Qiuyue Ran, Xiande Duan, Zhifang He, Shunqiang Yang, Muhammad Junaid Rao

**Affiliations:** 1 Key Laboratory of Edible Fungi Resources Innovation Utilization and Cultivation, College of Agronomy and Life Sciences, Zhaotong, 657000 China; 2 National Key Laboratory for Development and Utilization of Forest Food Resources, Zhejiang A & F University, Hangzhou, 311300 Zhejiang China

**Keywords:** *Leucocalocybe mongolica*, Rice growth promotion, Photosynthesis, Nitrogen metabolism, Hormone signaling, Sustainable agriculture

## Abstract

The fungal species *Leucocalocybe mongolica* has garnered attention due to its plant growth-promoting capabilities without fertilizers and emerged as a significant subject of research offering promising applications in sustainable agricultural practices. This study investigated the effects of LY9-transformed soil on rice growth and development through physiochemical, phenotypic, transcriptomic, and metabolomic analyses. Soil treated with varying concentrations of LY9 (10%, 30%, and 50%) exhibited significant improvements in nutrient availability compared to untreated controls. Rice plants grown in LY9-transformed soil enhanced phenotypic characteristics, including increased tillering (up to 20.29 tillers vs. 9 in control), greater root length (52.5 cm vs. 42 cm), and elevated chlorophyll content (1.21 mg/g vs. 0.38 mg/g). Transcriptomic analysis revealed significant alterations in genes related to primary and secondary metabolism, with 2,612 upregulated and 3,419 downregulated genes. KEGG pathway analysis highlighted modifications in nitrogen metabolism (24 genes), photosynthesis (41 genes), hormone signaling and tillering (222 genes), and cell wall and amino acids biosynthesis (365 genes). LC-MS/MS metabolomic profiling identified substantial increases in key amino acids, alkaloids, and phytohormones in LY9-treated rice roots. Notably, tryptophan and its derivatives showed more than 2-fold increases, suggesting enhanced auxin biosynthesis potential. The study revealed intricate molecular mechanisms underlying LY9-mediated growth promotion, particularly through modulation of nitrogen metabolism and hormone signaling pathways. These findings demonstrate the potential of LY9 as a sustainable soil amendment for improving rice productivity and provide valuable insights into the molecular basis of plant-fungal interactions in agricultural systems.

**Supplementary Information:**

The online version contains supplementary material available at 10.1186/s12284-025-00813-4.

## Introduction

Rice (*Oryza sativa* L.) is a staple food crop that feeds more than half of the global population and plays a crucial role in food security worldwide (Fahad et al. 2019; Nawaz et al. 2022). However, declining soil fertility, environmental stresses, and the increasing demand for sustainable agricultural practices pose significant challenges to rice production (Fahad et al. 2019; Hashim et al. 2024). In recent years, the application of beneficial soil microorganisms and their derivatives has emerged as a promising strategy to enhance crop productivity while maintaining ecological balance (Njira 2013; Bargaz et al. 2018).

Through years of field surveys investigating various fairy rings caused by different fungi, we have identified a unique ecological phenomenon dominated by *Leucocalocybe mongolica* (LM), a wild edible mushroom with significant genetic peculiarities. Due to its characteristic gene loss, LM can only exist symbiotically within fairy rings, particularly those distributed across the inner Mongolia steppe region of China. These rings have demonstrated the remarkable ability to enhance pasture production, specifically of *Leymus chinensis*, without the need for fertilization (Duan et al. 2021). The growth-promoting effects are most pronounced in the DARK zone of the fairy rings, where plants exhibit darker green leaves and increased biomass compared to areas outside the rings (Duan et al. 2022b). Among the fungi studied, *Leucocalocybe mongolica* has emerged as a species of particular interest due to its potential growth-promoting properties, though its effects on crops like rice remain largely unexplored (Duan et al. 2022a, b).

Soil health and fertility are fundamental factors determining crop productivity and sustainability in agricultural systems (Biswas et al. 2014; Tahat et al. 2020). The complex interactions between soil physicochemical properties, plant root development, and nutrient uptake mechanisms significantly influence plant growth and yield potential (Dunbabin et al. 2013; Jin et al. 2017; Kolb et al. 2017; Duan et al. 2024). Traditional approaches to improving soil fertility often rely heavily on chemical fertilizers, which can lead to environmental degradation and reduced soil quality over time (Verma et al. 2019; Jatav et al. 2021). Therefore, there is an urgent need to develop alternative, environmentally friendly strategies to enhance soil fertility and crop productivity.

Recent studies have shown that certain fungal species can significantly modify soil properties and enhance plant growth through various mechanisms (Adedayo and Babalola 2023). These include improvements in soil structure, enhanced nutrient availability, and the production of bioactive compounds that stimulate plant growth and development (Odoh et al. 2020). *Leucocalocybe mongolica* (forming typical fairy rings), a basidiomycete fungus native to Asian grasslands, has shown promising results in preliminary studies for its potential to enhance soil fertility and promote plant growth without fertilization (Yang et al. 2019; Duan et al. 2022a, b; Wang et al. 2022). However, the detailed mechanisms underlying their effects on soil properties, plant physiological and molecular responses, particularly in rice cultivation, remain to be fully elucidated. Understanding the molecular mechanisms and metabolic pathways involved in plant responses to beneficial soil organisms is crucial for developing effective agricultural applications (Hakeem and Akhtar 2016). Recent advances in transcriptomic and metabolomic analyses have provided powerful tools to investigate these complex interactions at the molecular level (Yang et al. 2021; Mahmood et al. 2022). Such comprehensive approaches can reveal key insights into how soil amendments influence plant growth, development, and stress responses through changes in gene expression patterns and metabolite profiles.

Strain LY9 is a microbial strain that was carefully isolated and cultured from the fruiting body of *Leucocalocybe mongolica* (LM). By conducting fermentation with a soil-based substrate, our aim is to artificially mimic the plant - growth - promoting properties inherent in the wild LM fairy - ring ecosystem. Considering that rice and *Leymus chinensis* both fall within the Poaceae family and exhibit comparable biological morphologies; we are eager to explore the application of the LY9 strain for enhancing rice growth. This study investigated the effects of different concentrations of LY9-transformed soil on rice growth and development, focusing on changes in soil physicochemical properties, plant phenotypic responses, and underlying molecular mechanisms. We employed a multi-omics approach, combining detailed soil analysis, phenotypic characterization, transcriptomics, and metabolomics to provide a comprehensive understanding of how LY9 treatment influences rice plant growth and development. Our research specifically aimed to evaluate the effects of LY9-transformed soil on rice root development, tillering capacity, and overall plant growth. We elucidate the molecular mechanisms underlying the observed growth responses through transcriptomic and metabolomic analyses and identify key metabolic pathways and signaling networks involved in LY9-mediated growth promotion. This study provides valuable insights into the potential applications of LY9 strain in sustainable rice cultivation and has significant implications for developing environmentally friendly approaches to enhance crop productivity and soil health in sustainable agriculture.

## Materials and Methods

### Planting Materials and Growing Conditions

#### Cultivation of LY9 Strain

The LY9 strain was isolated from *Leucocalocybe mongolica* fruiting bodies and authenticated via ITS sequencing (GenBank accession no. PP496285.1). The live strain of the model is preserved in the Guangdong Microbial Culture Collection Center (Accession Number: 63678). In this experiment, potato dextrose agar (PDA) medium was prepared (Supplementary File 1) and the strain LY9 was inoculated and cultured until mycelial growth covered the medium, providing the material basis for transformed soil preparation. Under sterile conditions, a small piece of LY9 strain was transferred using a sterile inoculation spatula onto PDA medium, with the mycelium positioned face-down on the substrate surface. The culture plates were incubated at 25 °C for 20 days until complete mycelial coverage was achieved (Supplementary Fig. 1).

## Preparation of LY9 Transformation Soil

For LY9 transformation soil preparation, mycelium grown on PDA medium was used to generate solid-state cultures, which subsequently served as inoculum for rice cultivation experiments. High-quality wheat grains were thoroughly washed until the rinse water ran clear and soaked for 12 h. The prepared grains were placed in polyethylene bags (22 cm × 45 cm) and autoclaved at 121 °C for 60 min. After cooling, the grains were inoculated with LY9 culture from PDA medium and incubated for 40 days until complete colonization.

The growth substrate consisted of paddy field soil (obtained from local dry paddy fields) and peat soil (Pin’s peat soil) mixed in a 1:1 ratio. This mixture was sterilized in polyethylene bags fitted with inoculation rods at 121 °C for 60 min. Subsequently, approximately 10 g of LY9-colonized wheat grain spawn was inoculated into each bag. The bags were incubated at room temperature in darkness for 60 days until complete mycelial colonization was achieved. The process yielded 100 bags of LY9 transformation soil, totaling approximately 150 kg wet weight (Supplementary Fig. 2a). The fully colonized substrate appeared uniformly white (Supplementary Fig. 2b).

## LY9 Transformation Soil and Rice Growth Experiment

The effects of LY9-transformed soil on rice (*Oryza sativa* ssp. japonica cv. ZH11) growth using an outdoor potting system. Different proportions of LY9-transformed soil were evaluated for phenotypic assessment and tissue sampling.

Rice plants were grown in outdoor pots (25 cm × 35 cm) and LY9-transformed soil was mixed with paddy soil at ratios of 10%, 30%, and 50%, with untreated paddy soil (0%) serving as control (CK). Transplantation and outdoor cultivation were initiated on June 15, 2023. No additional fertilization was applied during the experimental period. For phenotypic assessments, 10 technical replicates per treatment while three independent biological replicates were used for molecular analyses (transcriptomics, metabolomics). Sampling of root tissues was conducted at 90 days post-transplantation, coinciding with the tillering stage.

**Fig. 1:**
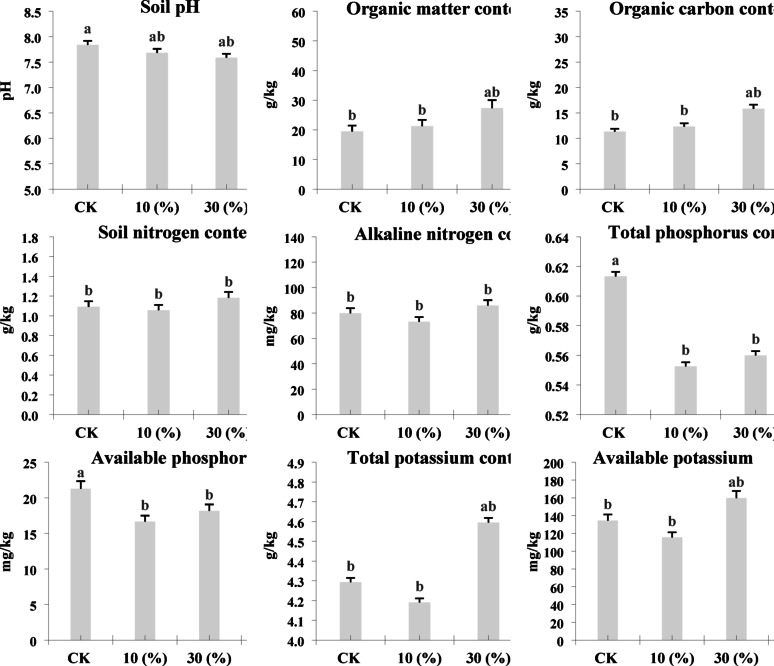
Illustrates the physicochemical parameters among control vs. LY9-treated (e.g., CK vs. 10%, 30%, 50%). The parameters include soil pH, organic matter content, organic carbon content, total nitrogen content, alkaline nitrogen levels, available phosphorus, total phosphorus, total potassium and available potassium. The data points presented in each column represent the mean values derived from three independent biological replicates. Statistical analysis was performed using the least significant difference (LSD) test to determine significant variations between different samples (*p* < 0.05). Different lowercase letters (a, b, c) indicate statistically significant differences between treatments

After approximately two months of cultivation, plants grown in LY9-transformed soil exhibited significant growth promotion compared to controls, as demonstrated in Fig. 1. To further investigate the growth-promoting effects of LY9-transformed soil, rice samples were collected from plants grown in CK, 10%, 30%, and 50% treatments using liquid nitrogen flash-freezing, for further analysis.

## Biochemical Parameters of Soil

Soil pH was determined using the glass electrode method (Bargrizan et al. 2017). Air-dried soil (10 g, 2 mm sieved) was mixed with CO₂-free distilled water (2.5:1 water-to-soil ratio), stirred for 1 min, and allowed to settle for 30 min before measurement with a calibrated pH meter (FE28, METTLER-TOLEDO).

Total organic carbon and organic matter were quantified via the potassium dichromate oxidation-external heating method (Hou et al. 2023). Soil (0.25 g, 0.25 mm sieved) was digested with 0.4 mol/L K₂Cr₂O₇-H₂SO₄ at 180 °C for 5 min, and residual dichromate was titrated with FeSO₄. Organic carbon was calculated as:

$$ \begin{gathered} {\text{Total organic carbon}}\left( {g/kg} \right) \hfill \\ = \left[ {(c \times \left( {V_{0} {\mkern 1mu} - {\mkern 1mu} V} \right) \times 0.003 \times 1.10{\text{ }})/m} \right] \times 1000 \hfill \\ \end{gathered} $$

$$ \begin{aligned}   Organic{\text{ }}matter\left( {g/kg} \right) =  & \left[ {\left( {c \times (V_{0}  - V) \times 0.003} \right.} \right. \\     \times  & \left. {\left. {1.724 \times 1.10} \right)/m} \right] \times 1000 \\  \end{aligned}  $$

where *V*_*0*_ and V are FeSO₄ volumes (mL) for blank and sample, respectively, *c* is FeSO₄ concentration (mol/L), 1.10 is oxidation correction factor, 0.003 is 1/4 millimolar mass of carbon atom, in grams (g), 1000 is to convert the content into per kilogram, and *m* is sample mass (g). Organic matter was derived by multiplying organic carbon by 1.724 (coefficient for converting organic carbon to organic matter).

### Soil Nutrients

#### Soil Nitrogen Content

Total soil nitrogen content was measured using the Kjeldahl method (Bremner 1960). Soil (1.0 g, 0.148 mm sieved) was digested with H₂SO₄ and catalyst (K₂SO₄:CuSO₄:Se = 100:10:1) at 400 °C, distilled, and titrated with 0.01 mol/L H₂SO₄. Total soil nitrogen content was calculated as:

$$ \begin{gathered} {\text{Total soil nitrogen content}}\left( {g/kg} \right) \hfill \\ = [(V_{1} {\mkern 1mu} - {\mkern 1mu} V_{0} ) \times c \times 14.01 \times D \times 1000/m]/ \times 0.001 \hfill \\ \end{gathered} $$

where V_1_ is the volume of sulfuric acid standard titration solution consumed by the test solution in milliliters (mL), V_0_ is the volume of sulfuric acid standard titration solution consumed by the reagent blank in mL, c is the concentration of sulfuric acid standard titration solution in moles per liter (mol/L), 14.01 is the molar mass of nitrogen atom (g/mol), D is dilution factor, and *m* is the mass of dried soil sample (g).

Alkaline nitrogen was extracted with 1.2 mol/L NaOH and diffused into H₃BO₃ indicator solution for titration (Chen and Xu 2008), the titration of absorbed NH₃ with a 0.01 mol/L sulfuric acid (H₂SO₄) standard solution. The alkaline nitrogen content (mg/kg) was calculated using the formula:

$$ \begin{gathered} {\text{Alkaline nitrogen}}\left( {mg/kg} \right) \hfill \\ = \left[ {(c \times (V - V_{0}) \times 14.0)/m} \right] \times 10^{3} \hfill \\ \end{gathered} $$

where *c* represents the concentration of the H₂SO₄ standard solution (mol/L), *V* is the volume of H₂SO₄ consumed in the sample titration (mL), *V₀* is the volume of H₂SO₄ consumed in the blank titration (mL), 14.0 is the molar mass of nitrogen (g/mol), *m* is the mass of the soil sample (g), and 10³ is the unit conversion factor.

#### Soil Phosphorus Content

Total phosphorus and available phosphorus were analyzed via NaOH fusion-molybdenum antimony spectrophotometry and NaHCO₃ extraction, respectively (Hedley et al. 1982). Total phosphorus was calculated as:

$$ {\text{Total phosphorus}}\left( {g/kg} \right){\mkern 1mu} = {\mkern 1mu} C \times V \times D \times 10^{{ - {\kern 1pt} 3}} /m $$

where *C* is P concentration (mg/L) from the standard curve, *V* is the volume of color developing solution 25mL, *D* is the dispensing multiple fixed volume/dispensing volume, 10^−3^ is the conversion factor for converting the unit mL to L, and *m* is the weighed sample mass (g).

Available phosphorus was extracted with 0.5 mol/L NaHCO₃ (pH 8.5) and quantified similarly. The following formula was used to determine the available phosphorus (AP) content in soil (mg/kg):

$${\text{Total available phosphorus}}\left( {g/kg} \right)=(r\, \times \,V \times ts)/m$$

Where *ρ* is the mass concentration of P obtained from the working curve (µg/mL); *V* is the fixed volume during color development (mL), *ts* is to take multiples, and *m* is the air-dried soil mass (g).

#### Soil Potassium Content

Total potassium and soil available potassium were determined by NaOH fusion-flame photometry and NH₄OAc extraction, respectively (Zebec et al. 2017). Total potassium was calculated as:

$$ \begin{gathered} {\text{Total potassium}}\left( {g/kg} \right) \hfill \\ = (C \times V \times D \times 10^{{ - 3}} )/m \hfill \\ \end{gathered} $$

Where: *C* is the potassium concentration in the test solution obtained from the calibration curve or regression equation (mg/L), *V* is the extraction volume (50mL), *D* is the dilution multiple, *m* is the weighed sample mass (g), and 10^−3^ is the conversion factor. Available potassium was extracted with 1 mol/L NH₄OAc (pH 7.0) and measured via flame photometer (FP6450). Soil available potassium was calculated as:

$$ \begin{gathered} {\text{Soil available potassium}}\left( {g/kg} \right) \hfill \\ = C \times \left( {V/m} \right) \times f \hfill \\ \end{gathered} $$

Where: C is the mass concentration of K in mg/L, *V* is the mL of added leaching agent, *m* is the mass of dried soil sample (g), and f is the dilution factor.

### RNA Sequencing of Rice Root Tissues

Total RNA was extracted from rice roots using Trizol reagent (Takara-kit). RNA-sequencing libraries were constructed using the UltraTM RNA Library Prep Kit according to the manufacturer’s protocol (NEB, USA). The libraries underwent paired-end sequencing on an Illumina HiSeq 2500 platform. Following the removal of adaptor sequences and low-quality reads, the clean reads were mapped to the rice reference genome (IRGSP-1.0_genome.fa, accessed from https://rapdb.dna.affrc.go.jp/index.html on July 03, 2024). Gene function annotation was performed using multiple databases according to established protocols. Quality assessment of RNA-seq raw data was conducted using FastQC (Brown et al. 2017), followed by adaptor trimming with Cutadapt (Martin 2011). The processed reads were aligned to the reference genome using HISAT2. Differentially expressed genes (DEGs) were identified using Cuffdiff, with significance thresholds of|Log2FoldChange| ≥1 and P-value < 0.05. All rice root DEGs are provided in the supplementary Table 2. Gene enrichment analysis of DEGs was performed according to standard protocols (Zheng and Wang 2008), and KOBAS software was utilized to determine statistical enrichment of the identified DEGs.

### Metabolome and Hormonal Profiling of Rice Root

The metabolic and hormonal profiling of rice root samples was performed using ultra-performance liquid chromatography coupled with tandem mass spectrometry (UPLC-MS/MS). Sample preparation began by pulverizing freeze-dried tissue into a homogeneous powder, from which 50 mg aliquots were isolated for metabolic analysis. The extraction was conducted using 500 µL of a solution containing 50% methanol and 0.1% HCl (Chen et al. 2013). The samples were subjected to sonication for 30 min, followed by centrifugation at 12,200×g for 5 min at ambient temperature. Prior to instrumental analysis, the supernatant was filtered through a 0.22 μm membrane. UPLC-MS/MS analysis was performed following the established protocols described earlier (Chen et al. 2013). Metabolite identification was achieved through comparative analysis of precursor ions (Q1), product ions (Q3), characteristic fragmentation patterns, and retention times against authenticated reference standards analyzed under identical conditions (Chen et al. 2013). All analyses were conducted in triplicate, and data processing was performed using Analyst 1.6.3 software. A comprehensive detail of hormones and metabolic compounds (including Q1, Q3, characteristic fragmentation patterns, etc.,) are provided in Supplementary Table 1.

### Total Chlorophyll Contents (mg/g)

Flag rice leaf samples cultivated in LY9-transformed soil (control, 10%, 30%, and 50% treatments) were analyzed in triplicate for total chlorophyll content. For each sample, 0.2 g of leaf tissue was homogenized in a mortar with quartz sand, calcium carbonate powder, and 10 mL of 95% ethanol. An additional 5 mL of 95% ethanol was added during grinding until the tissue became colorless. After standing for 3–5 min, the homogenate was filtered into a 25 mL amber volumetric flask. The residue on the filter paper was rinsed with 95% ethanol, and the final volume was adjusted to 25 mL with ethanol. The absorbance of the extract was measured at 665 nm and 649 nm using a spectrophotometer with a 1 cm path length cuvette, using 95% ethanol as a blank. Chlorophyll concentrations were calculated using the following equations:

$$ \begin{gathered} {\text{Chlorophyll a }}\left( {{\text{mg}}/{\text{g fresh weight}}} \right) \hfill \\ = {\text{Chlorophyll a}} \times {\text{V}} \times {\text{N}}/({\text{W}} \times {\text{1}}000) \hfill \\ \end{gathered} $$

$$ \begin{gathered} {\text{Chlorophyll b}}\left( {{\text{mg}}/{\text{g fresh weight}}} \right) \hfill \\ = {\text{Chlorophyll b}} \times {\text{V}} \times {\text{N}}/({\text{W}} \times {\text{1}}000) \hfill \\ \end{gathered} $$

$$ $$$$\begin{gathered} {\text{Total chlorophyll}}\left( {{\text{mg}}/{\text{g fresh weight}}} \right) \hfill \\ = {\text{Chlorophyll a}} + {\text{Chlorophyll b}} \hfill \\ \end{gathered} $$

Where: V = extract volume (mL); N = dilution factor; W = fresh tissue weight (g).

### Statistical Analysis

A multivariate statistical analysis was performed on the metabolomic and transcriptomic datasets. Prior to hierarchical cluster analysis (HCA), data was normalized (Z-score) for both transcriptomic and metabolomic analyses. Statistical evaluation included HCA and principal component analysis (PCA) following established protocols (Chanana et al. 2020). Statistical significance of variations in physiochemical properties and morphological parameters were determined using the least significant difference test to determine the variations between different samples (*p* < 0.05). Standard error calculations and statistical analyses were performed using Statistix (Tallahassee, FL, USA) software version 8.1, with all experiments conducted in triplicate.

## Results

### Soil Physicochemical Characteristics

Soil samples were transformed with three concentrations (10%, 30%, and 50%) of LY9, and detailed analyses of soil physicochemical characteristics were conducted, with untreated soil serving as the control. The soil pH showed a notable reduction from 7.84 in control conditions to 7.17 following 50% LY9 treatment (Fig. 1). Significant improvement in soil organic components was observed, with organic matter content increasing by approximately 149% (from 19.5 g/kg to 48.63 g/kg) and organic carbon content showing a similar upward trend from 11.31 g/kg to 28.21 g/kg under 50% LY9 treatment conditions (Fig. 1). The total nitrogen content increased from 1.09 g/kg to 1.53 g/kg, while alkaline nitrogen levels rose from 79.82 mg/kg to 117.1 mg/kg in 50% LY9-treated soils. Similarly, the total potassium and available potassium content showed a significant increment from 4.29 g/kg and 134 mg/kg in control conditions to 4.81 g/kg and 179 mg/kg in 50% LY9-treated soil, respectively, suggesting improved nutrient availability for rice plant uptake (Fig. 1). Interestingly, total phosphorus and available phosphorus content showed a slight but significant decrease from 0.61 g/kg and 21.3 mg/kg in control conditions to 0.55 g/kg and 17.6 mg/kg in 50% LY9-treated soil, respectively (Fig. 1). The results showed that LY9 treatment significantly enhances soil organic matter content and nitrogen availability, potentially through improved nutrient cycling mechanisms. These modifications in soil physiochemical properties create more favorable conditions for plant growth and development, suggesting potential applications in sustainable agriculture and soil improvement strategies.

### LY9-soil Improves Rice Root Growth, Enhances Tillering, and Green Leaf Phenotypes

**Fig. 2:**
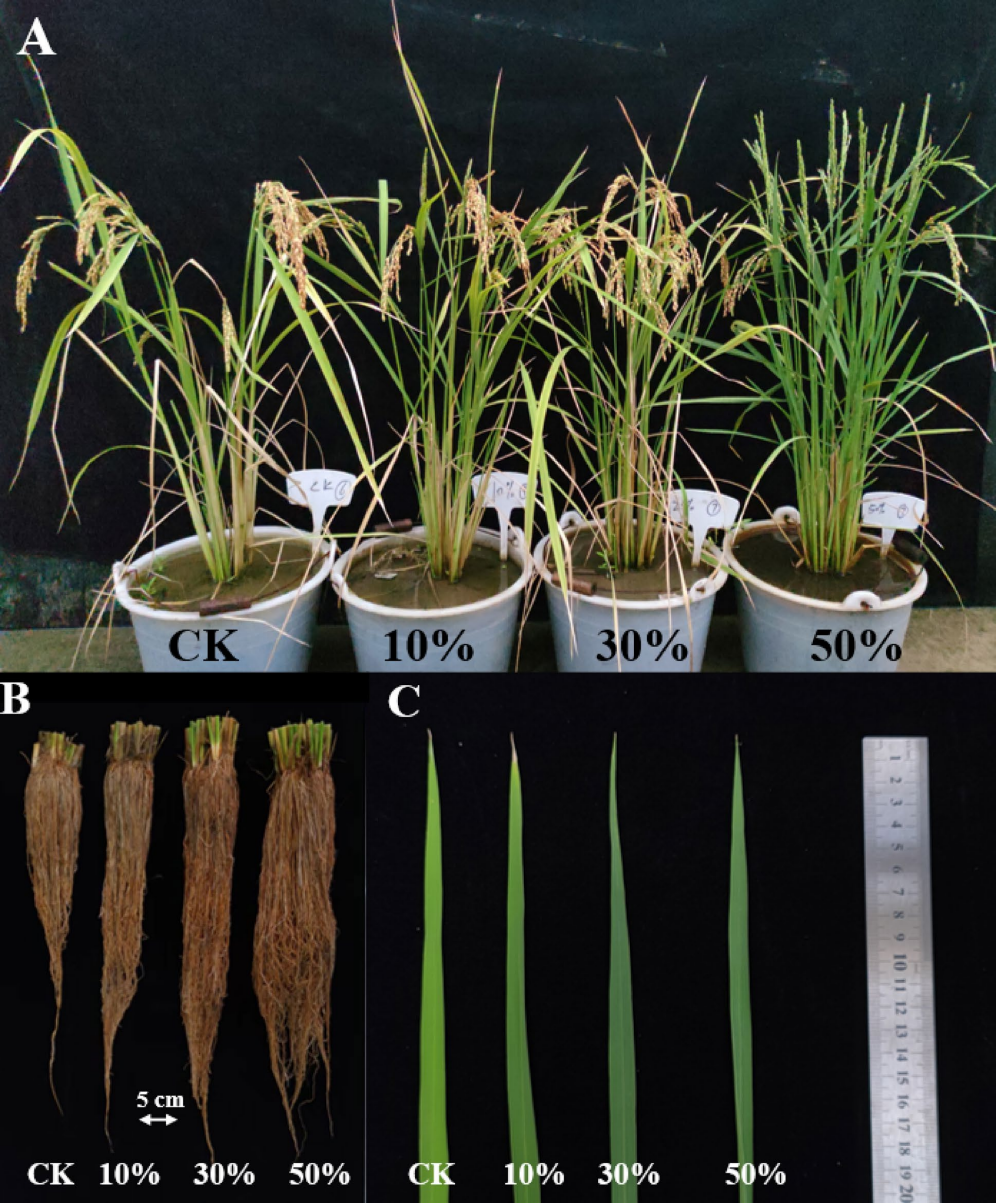
Growth-promoting effects on rice plants control vs. LY9-treated (e.g., CK vs. 10%, 30%, 50%). **A** Rice plants grown in LY9-transformed soil demonstrated enhanced tillering phenotype compared to CK, after 90 days of cultivation. **B** Root morphology comparison among treatments. **C** Leaf phenotypes of rice plants treated with LY9 exhibited significantly darker green coloration compared to CK leaves

Phenotypic analyses were conducted on rice plants cultivated under varying ratios of LY9 vs. CK (Fig. 2). The rice grown with LY9-transformed soil produced an increased number of tillering than control (Fig. 2A). The roots of rice grown with LY9-transformed soil were larger in length with enhanced lateral root formation and greater root biomass than rice roots grown under control conditions (Fig. 2B). The rice leaves harvested from LY9-transformed soil showed intensified green color phenotype indicating potentially elevated chlorophyll content and enhanced photosynthetic capacity, as compared to the lighter green leaves harvested from control rice plants (Fig. 2C). These phenotypic observations suggested that the LY9-transformed soil significantly influenced the rice plant growth and development, including enhanced root system architecture, increased tillering capacity, and improved foliar characteristics, resulting in more robust and vigorous plants than control rice plants. The observed morphological and physiological enhancements suggest that LY9-transformation of soil may trigger beneficial alterations in soil properties (Fig. 1), potentially leading to improved plant performance and productivity.

**Fig. 3:**
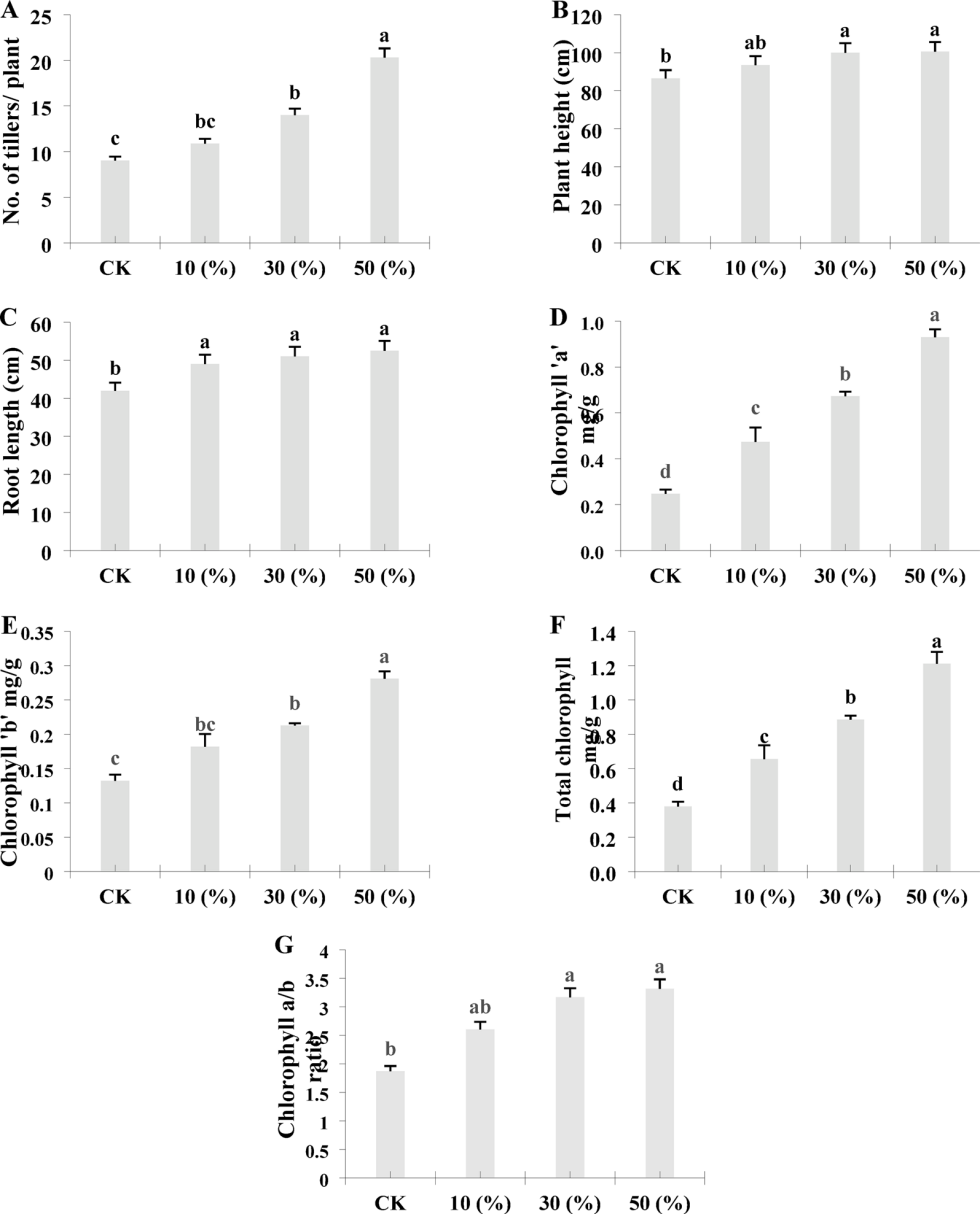
Morphological and physiological characteristics of rice plants under control vs. LY9-treated (e.g., CK vs. 10%, 30%, 50%). The growth parameters include **A** tillering capacity, **B** plant height, **C** root length, **D** chlorophyll ‘a’, **E** chlorophyll ‘b’, **F** total chlorophyll contents, and **G** chlorophyll a/b ratio. The data points presented in each column represent the mean values derived from three independent biological replicates. Statistical analysis was performed using the least significant difference (LSD) test to determine significant variations between different samples (*p* < 0.05). Different lowercase letters (a, b, c) indicate statistically significant differences between treatments

The incorporation of LY9-transformed soil significantly enhanced rice plant growth and developmental parameters. Soil treated with LY9 at ratios of 10%, 30%, and 50% significantly increased tiller numbers per plant to 10.86, 14, and 20.29 respectively, compared to 9 tillers per plant in control (Fig. 3A). The increased tiller numbers indicate potential for improved biomass accumulation compared to control rice plants. Plant height was significantly higher in the 50% LY9-transformed soil treatment, reaching 100.55 cm compared to 86.5 cm in controls (Fig. 3B). Similarly, root length increased significantly to 52.5 cm in plants grown in 50% LY9-transformed soil compared to 42 cm in controls (Fig. 3C). Chlorophyll a content was significantly elevated in 10%, 30%, and 50% LY9-transformed soil treatments, measuring 0.93 mg/g, 0.67 mg/g, and 0.47 mg/g respectively, compared to 0.25 mg/g in control leaves (Fig. 3D). Corresponding trends were observed for chlorophyll b content, which measured 0.28 mg/g, 0.21 mg/g, and 0.18 mg/g in the 10%, 30%, and 50% treatments respectively, compared to 0.13 mg/g in controls (Fig. 3E). Total chlorophyll content was 1.21 mg/g, 0.89 mg/g, and 0.66 mg/g in the respective LY9-transformed soil treatments, compared to 0.38 mg/g in controls (Fig. 3F). The rice roots harvested from 50%, 30%, and 10% LY9-treated soil showed significantly higher chlorophyll a/b ratio 2.61, 3.17, and 3.32 respectively, than control rice roots 1.87 (Fig. 3G). A higher chlorophyll a/b ratio in rice leaves typically indicates greater efficiency in light absorption for photosynthesis. These results showed that LY9-transformed soil promotes rice plant growth and development with improved chlorophyll content suggesting enhanced light-harvesting capabilities and improved photosynthetic capacity. Based on these promising preliminary findings, we conducted detailed transcriptomic analysis of root tissues to elucidate the molecular mechanisms underlying these growth-promoting effects in LY9-treated rice plants.

### Transcriptomic Analysis of Rice Roots

**Fig. 4:**
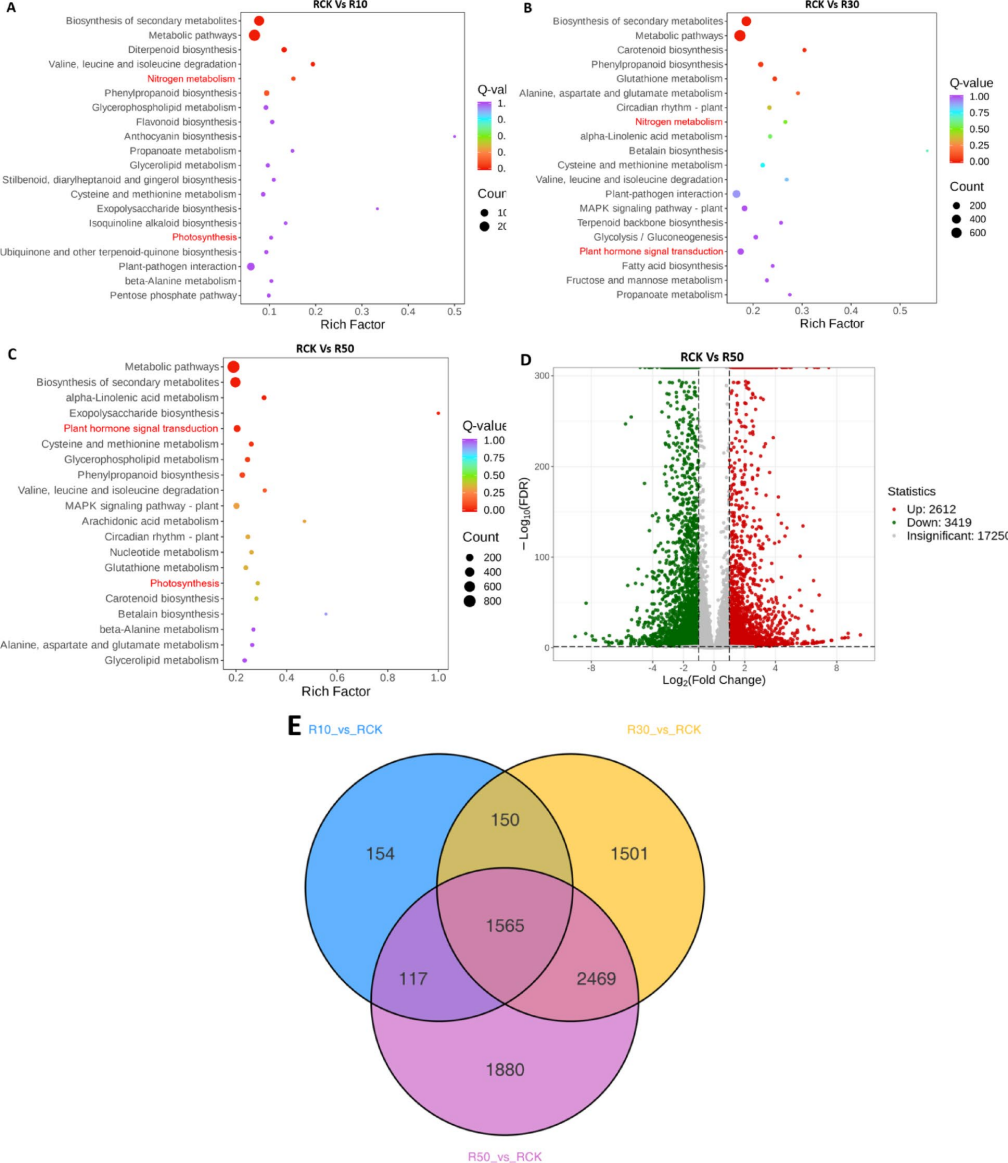
Root transcriptomic analysis revealed significant gene expression changes in rice roots control vs. LY9-treated (e.g., CK vs. 10%, 30%, 50%). **A** KEGG pathway enrichment analysis (CK vs. 10%). **B** KEGG pathway enrichment analysis (CK vs. 30%). **C** KEGG pathway enrichment analysis (CK vs. 50%). **D** Volcano plot illustrates differential gene expression patterns (CK vs. 50%). **E** Venn diagram depicting the overlap of differentially expressed genes in different treatment groups, R: root

A KEGG enrichment analysis was performed to determine the differential metabolite categories regulated in rice roots grown in LY9-treated soil (10%, 30%, and 50%) compared to the CK (Fig. 4). The analysis revealed that secondary metabolite biosynthesis and metabolic pathway genes were most significantly affected in rice roots exposed to LY9-treated soil. The impact on metabolic biosynthesis genes increased proportionally with higher concentrations of LY9-treated soil (10–50%). Furthermore, KEGG enrichment analysis demonstrated significant alterations in nitrogen metabolism, photosynthesis, and plant hormone signal transduction genes in LY9-treated rice compared to CK (Fig. 4A-C). These modifications in hormone and photosynthesis-related genes directly influenced rice plant growth and development, correlating with the observed phenotypic differences between LY9-treated and control plants (Fig. 2A-C). Some other metabolism pathway genes such as phenylpropanoid pathway, amino acid biosynthesis/degradation pathway, isoquinoline alkaloid biosynthesis, and glutathione metabolism were also significantly altered in KEGG enrichment analysis (Fig. 3A-C). Differential gene expression analysis of rice roots, visualized through a volcano plot, identified 2,612 upregulated genes and 3,419 downregulated genes, while 17,250 genes showed no significant changes (Fig. 2D).

The root Venn analysis revealed that 1,565 genes were commonly regulated across all R10, R30, and R50 LY9-treated rice samples compared to RCK controls. Differential gene expression analysis showed 154, 1,501, and 1,880 genes were uniquely regulated in RCK vs. R10, RCK vs. R30, and RCK vs. R50 comparisons, respectively. This demonstrates that increasing concentrations of LY9 resulted in larger numbers of differentially expressed genes (Fig. 2E). The transcriptomic analysis highlights significant number of genes that differentially altered associated with cell wall and amino acid (365 genes), hormonal signaling and tillering (222 genes), photosynthesis (41 genes), nitrogen metabolism (24 genes), and alkaloids (10 genes) (Supplementary Table 3). Therefore we performed detailed LC-MS/MS analysis focusing on hormones, alkaloids and amino acids in rice roots to find out which metabolites are responsible for enhanced rice growth characteristics, tillering capacity, and intensified green color in LY9-treated rice plants.

### Primary and Secondary Metabolic Pathway Genes, Tillering, and Hormone Signaling Transduction Pathway Genes Were Significantly Upregulated in LY9 Rice Roots

Transcriptomic analysis revealed complex molecular mechanisms underlying the response to rice grown in LY9-treated soil. The expression profiles were significantly upregulated by multiple gene families involved in primary and secondary metabolism (Table 1). Specifically, genes associated with sugar and carbohydrate metabolism (Os05g0426000) and pentose phosphate pathway-related gene Os06g0114651 exhibited enhanced expression levels by more than 10-folds (Table 1). Notable increases were also observed in genes controlling amino acid biosynthesis and metabolism (Os02g0120100, Os02g0482400), suggesting enhanced protein synthesis capacity. The photosynthetic machinery components, particularly chloroplast-associated genes (Os01g0322700), displayed significant upregulation, indicating improved photosynthetic efficiency. Furthermore, genes involved in nitrogen metabolism and alkaloid biosynthesis (Os01g0977300) showed significant increment, along with hormone signaling cascade components Os06g0692500, including auxin-responsive elements Os01g0977300 and gibberellin metabolism regulators Os01g0332200 (Table 1). These genes showed more than 10-fold upregulation across all LY9 treatment concentrations (10%, 30%, and 50%) compared to control conditions. The analysis also revealed that tillering-associated genes, including Os07g0178700, Os02g0651300, and Os01g0595600, exhibited more than 2-fold increased expression in LY9-grown rice roots relative to controls, suggesting enhanced vegetative growth potential.

An interesting finding was the coordinated upregulation of various transcription factor families. The MYB44 transcription factor (Os01g0977300), known for its role in stress responses and secondary metabolism, showed significant upregulation (Table 1). Similarly, the basic helix-loop-helix transcription factor (Os02g0805250), LOB29 (Os03g0445700), and the sugar-responsive NAC48 (NAM) transcription factor (Os02g0214500) all demonstrated more than 4-fold increased expression across all LY9 treatment levels (Table 1). These regulatory elements play crucial roles in various physiological processes, including auxin response pathways, secondary metabolite production, nitrogen compound metabolism, hormonal signal transduction, organ morphogenesis, and sugar response mechanisms. Our expression data revealed that LY9-treated soil conditions triggered significant transcriptional reprogramming, particularly affecting genes involved in fundamental metabolic processes such as sugar metabolism, photosynthetic efficiency, amino acid biosynthesis, nitrogen metabolism, hormone signaling, and organ development. The observed changes in gene expression patterns provide strong molecular evidence for the positive effects of LY9 treatment on rice growth parameters, offering valuable insights into the underlying mechanisms of improved plant performance under these conditions.

**Table 1:** Illustrates the transcriptomic analysis of rice roots harvested and the values represent fold-change upregulation (based on FPKM) in rice roots control vs. LY9-treated (e.g., CK vs. 10%, 30%, 50%)

Serial No.	Gene ID	Gene Description	KEGG pathway	CK vs. 10%	CK vs. 30%	CK vs. 50%
1	**Os01g0322700**	Regulation of panicle branching and grain weight, Regulation of grain yield and photosynthesis	–	15.51	124.37	119.06
2	**Os02g0120100**	Serine/threonine/tyrosine protein kinase	ko04016: MAPK signaling pathway - plant; ko04075: Plant hormone signal transduction	25.41	70.79	107.31
3	Os05g0426000	Sugar and carbohydrate transporter activity	K15382 solute carrier family 50 (sugar transporter) SWEET1b	30.22	29.82	64.20
4	**Os06g0692500**	Hypothetical conserved gene.	ko04075: Plant hormone signal transduction	12.78	15.31	40.25
5	**Os01g0977300**	Like MYB-related protein Response to auxins, metabolites, nitrogen-containing compounds etc.	K09422 transcription factor MYB44	12.72	32.46	33.90
6	**Os09g0507100**	Plant-specific transcription factor Regulation of panicle development	squamosa promoter-binding-like protein SBP	5.04	16.93	32.69
7	Os11g0256900	S-adenosyl-L-methionine: salicylic acid carboxyl methyltransferase (SAMT)	K21485 anthranilate O-methyltransferase [EC:2.1.1.277]	4.23	8.55	18.69
8	**Os02g0805250**	Basic helix-loop-helix transcription factor, Regulation of cell size	ko04075: Plant hormone signal transduction	3.98	8.23	17.75
9	Os05g0405000	Chloroplastic pyruvate orthophosphate dikinase	ko00010: Glycolysis; ko00620: Pyruvate metabolism; ko00710: Carbon fixation in photosynthetic organisms.	4.70	10.59	17.26
10	**Os01g0138800**	Peptidase C26 domain containing protein. regulation of secondary shoot formation cellular amino acid & nitrogen compound metabolic process	–	4.83	10.19	16.84
11	**Os03g0445700**	LATERAL ORGAN BOUNDARIES DOMAIN (LBD) protein, Activates leaf morphogenesis GO:0009965, shoot system morphogenesis GO:0010016, plant organ morphogenesis GO:1,905,392.	K13945 LOB domain-containing protein 29	6.35	12.78	15.82
12	Os03g0255500	Similar to Phosphoenolpyruvate carboxykinase 4	ko00010: Glycolysis, ko00020: Citrate cycle (TCA); ko00620: Pyruvate metabolism; ko00710: Carbon fixation in photosynthetic organisms.	6.43	12.27	14.28
13	Os06g0114651	Hypothetical gene. K00851 gluconokinase	ko00030: Pentose phosphate pathway; ko01200: Carbon metabolism	18.28	23.10	14.30
14	**Os11g0644800**	Similar to Tyrosine/nicotianamine aminotransferases	ko00350: Tyrosine metabolism; ko00950: Isoquinoline alkaloid biosynthesis; ko00960: Tropane, piperidine and pyridine alkaloid biosynthesis	6.27	14.17	13.88
16	Os02g0214500	Sugar-responsive NAC48 (NAM) transcription factor, Regulation of sugar	–	4.37	4.79	13.43
17	Os09g0464066	Hypothetical protein.	ko00910: Nitrogen metabolism;	5.61	8.96	11.81
18	**Os02g0750100**	Similar to ATP synthase.	ko00190: Oxidative phosphorylation; ko00195: Photosynthesis;	6.43	11.33	11.60
19	Os06g0561000	Myo-inositol oxygen	ko00053: Ascorbate and aldarate metabolism; ko00562: Inositol phosphate metabolism;	5.81	10.12	11.34
20	Os05g0438600	Fructose-1,6-bisphosphatase class 1/Sedoheputulose-1,7-bisphosphatase domain containing protein.	ko00010: Glycolysis ko00030: Pentose phosphate pathway; ko00710: Carbon fixation in photosynthetic organisms.	6.71	8.64	11.16
21	**Os02g0482400**	Similar to Ornithine decarboxylase (EC 4.1.1.17)	ko00330: Arginine and proline metabolism; ko00480: Glutathione metabolism;	9.89	16.14	10.04
22	Os07g0577600	Similar to Type II chlorophyll a/b binding protein from photosystem I precursor.	ko00196: Photosynthesis - antenna proteins;	4.01	6.90	9.94
23	**Os01g0332200**	GA 2-oxidase2, GA metabolism	K04125 gibberellin 2beta-dioxygenase [EC:1.14.11.13]	9.79	16.30	9.81
24	**Os09g0346500**	Chlorophyll a/b binding protein, Circadian clock-controlled gene	K08912 light-harvesting complex II chlorophyll a/b binding protein 1 ko00196: Photosynthesis - antenna proteins;	4.08	11.02	9.49
25	Os03g0340500	Like Sucrose synthase (EC 2.4.1.13).	K00695 sucrose synthase [EC:2.4.1.13]	2.80	4.81	9.48
26	**Os07g0556200**	Rieske [2Fe-2 S] region domain containing protein.	ko00195: Photosynthesis; transferring electrons within cytochrome b6/f complex of photosystem II activity	3.18	7.51	9.47
27	**Os07g0178700**	Similar to Low molecular mass early light-inducible protein HV90, chloroplast precursor (ELIP), Chlorophyll A-B binding protein	–	2.00	15.75	8.18
28	**Os02g0651300**	Carotenoid accumulation Regulation of tiller number	–	2.0	3.29	2.73
29	**Os01g0595600**	Alpha/beta hydrolase fold-1 domain containing protein. HIGH-TILLERING DWARF 2, secondary shoot formation	–	2.7	2.86	2.36
30	**Os02g0671100**	F-box domain protein, Regulation of root growth	–	2.47	3.34	4.39
31	**Os04g0543900**	Like **glutamate dehydrogenase** 1	K00261 glutamate dehydrogenase (NAD(P)+) [EC:1.4.1.3] ko00220: Arginine biosynthesis; ko00250: Alanine, aspartate and glutamate metabolism; ko00910: Nitrogen metabolism	2.38	3.26	4.45
32	**Os03g0712800**	Cytosolic **glutamine synthetase**, Early stage of seed germination and grain filling under N deficient conditions	K01915 glutamine synthetase [EC:6.3.1.2]| (RefSeq) glutamine synthetase cytosolic isozyme 1–3 (A) ko00220: Arginine biosynthesis; ko00250: Alanine, aspartate and glutamate metabolism; ko00910: Nitrogen metabolism; ko01230: Biosynthesis of amino acids	1.78	3.08	2.69
33	**Os02g0610700**	Chlorophyll a/b binding protein, domain containing protein.	–	2.0	2.48	3.55
34	**Os07g0562700**	Light-harvesting chlorophyll a/b-binding (LHCB) protein, Regulation of photosynthesis and antenna proteins	K08914 light-harvesting complex II chlorophyll a/b binding protein 3| chlorophyll a-b binding protein of LHCII type III, chloroplastic (A)	–	2.11	3.54
35	**Os10g0419500**	Acireductone dioxygenase%2 C Promotion of crown/lateral root development%2 C Modulation of the root growth pattern	K08967 1,2-dihydroxy-3-keto-5-methylthiopentene dioxygenase [EC:1.13.11.53 1.13.11.54]| 1,2-dihydroxy-3-keto-5-methylthiopentene dioxygenase 4 (A)	–	2.0	3.48
36	**Os11g0242400**	Light-harvesting chlorophyll a/b-binding (LHC) protein, Ortholog of AtPsb33.	–	2.08	3.97	3.35
37	**Os01g0227100**	Chlorophyll b reductase	K13606 chlorophyll(ide) b reductase [EC:1.1.1.294]| probable chlorophyll(ide) b reductase NYC1, chloroplastic (A)	2.02	3.18	2.99

The gene IDs which are bold means they are related to photosynthesis, chlorophyll a/b binding protein, cell elongation/size, plant organ morphogenesis, amino acid biosynthesis, nitrogen containing compounds metabolism, alkaloid metabolism, hormone signal transduction, and panicle branching/growth.

### Metabolic Profiling in LY9 Rice Roots

**Fig. 5:**
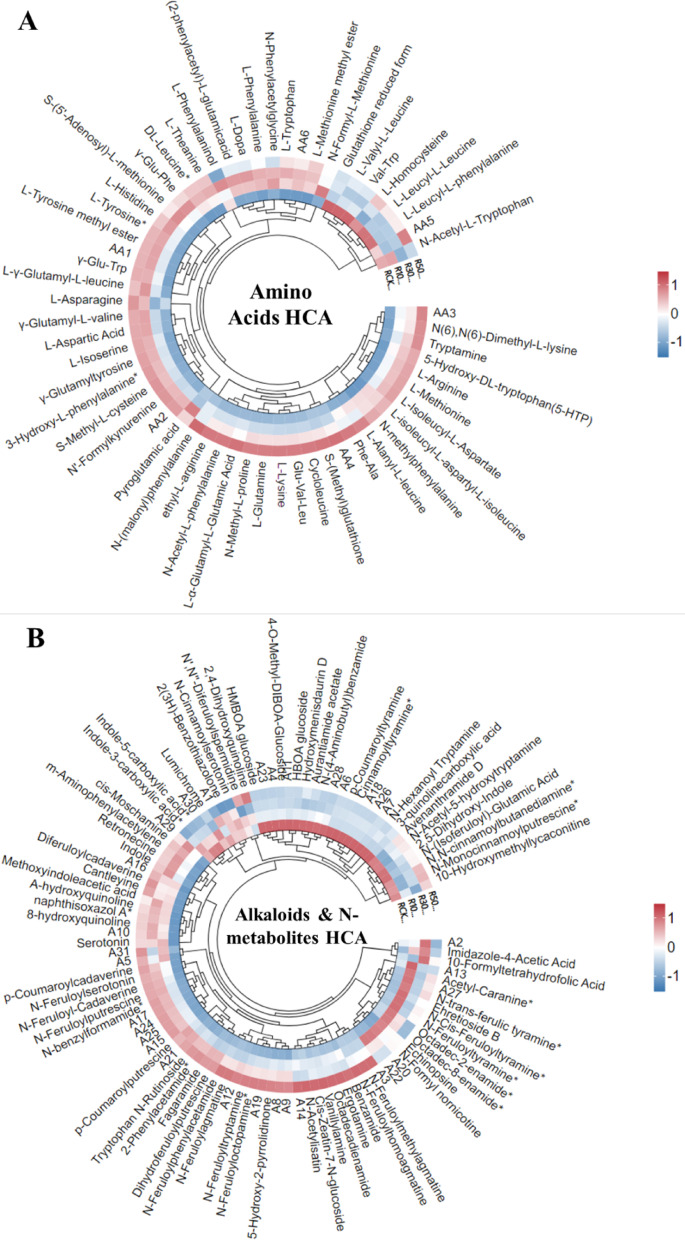

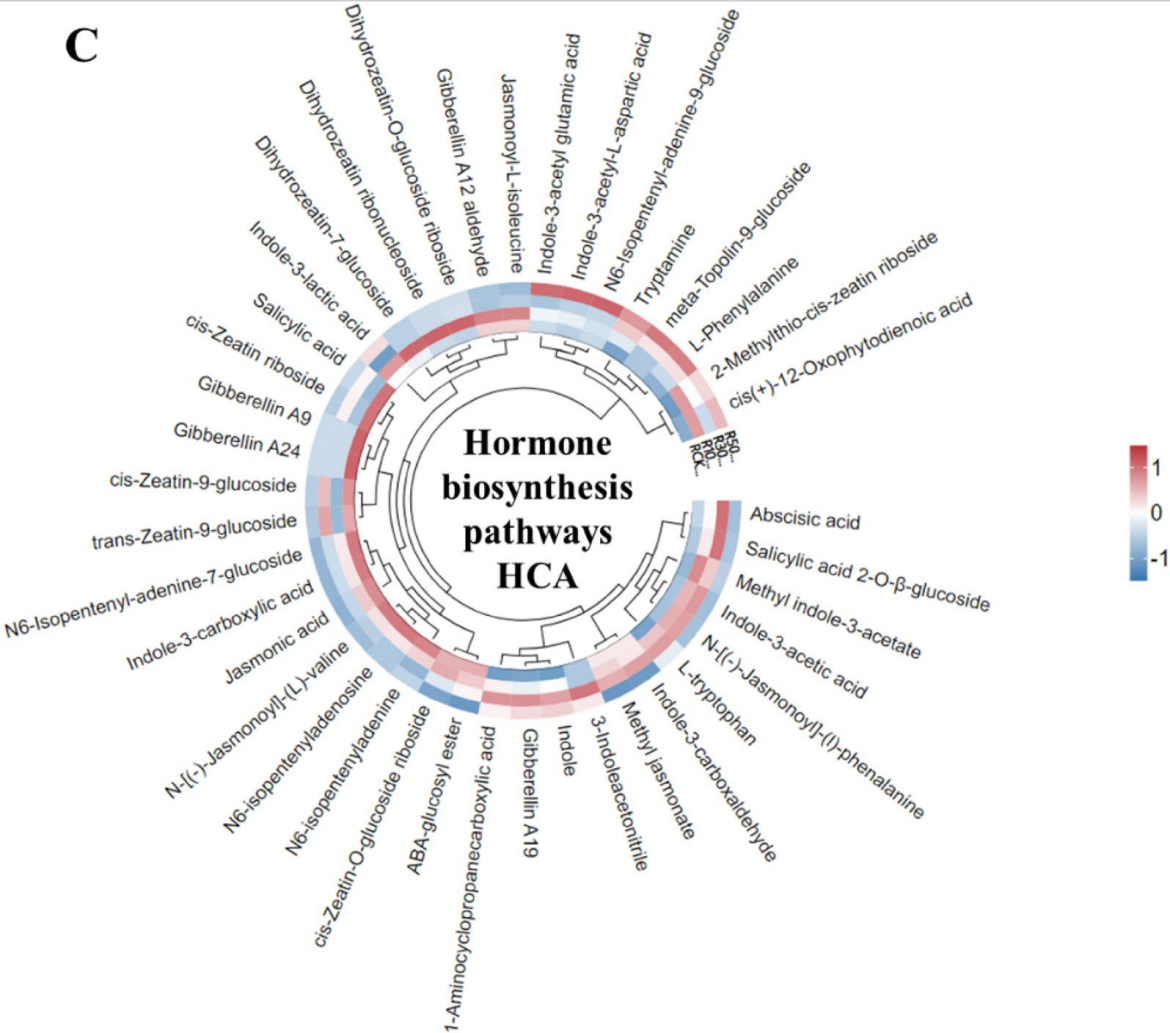
HCA analysis of rice root metabolites control vs. LY9-treated (e.g., CK vs. 10%, 30%, 50%). **A** Nitrogen containg compounds HCA including alkaloids, **B** Amino acids and their derivative HCA in rice roots, **C** Hormones and their pathways associated metabolites HCA in rice roots. The red color indicates high abundance of particular metabolite while the blue color represents lower abundance

Transcriptomic analysis revealed significant alterations in genes and pathways associated with amino acid biosynthesis, nitrogen metabolism, and hormonal regulation in rice roots harvested from LY9-treated soil (Fig. 4). Consequently, we conducted LC-MS/MS analysis to evaluate the abundance of amino acids, alkaloids, and hormones present in the rice root tissues. Our metabolomic analysis revealed significant alterations in amino acid profiles between LY9-treated and control rice roots (Fig. 5A). The data demonstrated an increasing trend of amino acids in LY9-treated samples, with only six amino acids showing decreased levels: L-valyl-L-leucine, Val-trp, L-homocysteine, L-leucyl-L-leucine, and L-leucyl-L-phenylalanine (Fig. 5A). Most notably, several key amino acids exhibited more than two-fold increment in the LY9-treated rice roots than control rice roots, including glutamine, arginine, tryptophan, tryptamine, methionine, histidine, aspartic acid, lysine, tyrosine, and L-Dopa (Fig. 5A). These elevated amino acids serve as crucial precursors for various bioactive compounds, including phytohormones, alkaloids, and polyphenolic compounds, which play pivotal roles in plant growth, development, and stress response mechanisms.

LC-MS/MS profiling identified 100 nitrogen-containing compounds, including various alkaloids, in the rice root samples (Fig. 5B). The HCA analysis revealed that 29 compounds showed downregulation in LY9-treated rice roots, while 71 compounds were significant upregulation compared to control conditions (Fig. 5B). Several compounds exhibited significant increases exceeding 4-fold in LY9-treated roots, including dihydroferuloylputrescine, fagaramide, indole-3-acetyl-L-aspartic acid, N’,N’’,N’’’-p-Coumaroyl-cinnamoyl-caffeoyl spermidine, N,N-dimethyl-5-methoxytryptamine, N-acetylisatin, and various N-feruloyl derivatives such as N-feruloyl-cadaverine, N-feruloylagmatine, N-feruloylhomoagmatine, N-feruloylmethylagmatine, N-feruloylputrescine, and its glucoside form (Fig. 5B). Additional compounds showing similar upregulation trend included N-feruloylserotonin, N-feruloyltryptamine, hexanediamine derivatives, tryptophan N-rutinoside, vanillylamine, cis-moschamine, and p-coumaroyl derivatives in the LY9-treated rice roots than control rice roots (Fig. 5B). Conversely, 14 compounds exhibited significant downregulation, exceeding 4-fold reduction in LY9-treated roots (Fig. 5B). These profound metabolic alterations in nitrogen-containing compounds correlated with enhanced root development and increased tillering phenotypes compared to control conditions.

### Hormonal Significantly Altered in Rice Roots

Phytohormone analysis revealed complex alteration in the hormones in response to LY9 treatment (Fig. 5C). Salicylic acid 2-O-β-glucoside and abscisic acid levels showed a biphasic response, with increased concentrations in roots grown under 10% and 30% LY9-treated soil, but decreased levels under 50% LY9 treatment compared to control rice roots (Fig. 5C). Several indole derivatives, including indole-3-acetyl glutamic acid, indole-3-lactic acid, and 3-indoleacetonitrile, demonstrated elevated levels specifically in roots harvested from 50% LY9-treated soil (Fig. 5C). Gibberellin A19 exhibited consistent upregulation across all LY9 treatment concentrations, indicating a robust hormonal response. The analysis of auxin metabolism revealed an interesting pattern: while indole-3-acetic acid (IAA) showed slightly upregulation (less than 2-fold increase) in LY9-treated roots, its precursor molecules, tryptophan and indole, demonstrated obvious increases exceeding 2-fold across all LY9 treatment levels (Fig. 5C). This differential regulation of auxin biosynthesis pathway and gibberellin A19 suggests a complex regulatory mechanism governing hormone homeostasis, which is positively correlated with enhanced root development and increased tillering phenotypes observed under LY9 treatment conditions. These findings provide crucial insights into the molecular mechanisms underlying plant adaptation to LY9 exposure and highlight the intricate interplay between various hormonal pathways in mediating growth responses.

The hormonal profiling of rice roots grown in LY9-transformed soil revealed significant alterations in phytohormones and their associated metabolites (Table 2). Notably, tryptophan, a precursor for auxin biosynthesis, exhibited a 2.16-fold increase in the 10% LY9 treatment, while tryptamine levels surged by 4.06-fold (Table 2). Indole, another key intermediate in auxin synthesis, also showed a 2.19-fold upregulation. Gibberellin A19, a growth-promoting hormone, increased by 3.50-fold in the 10% treatment, with even higher increments observed in the 30% (6.49-fold) and 50% (4.85-fold) treatments (Table 2). Conversely, cytokinin-related compounds such as N6-isopentenyladenosine and N6-isopentenyladenine were downregulated, suggesting a shift in hormonal balance favoring auxin and gibberellin pathways (Table 2). Additionally, salicylic acid 2-O-β-glucoside and abscisic acid displayed concentration-dependent responses, with initial increases in lower LY9 treatments but declines at higher concentrations (Table 2). These findings highlight the profound influence of LY9 on hormonal homeostasis, particularly in pathways linked to root development and tillering.

**Table 2:** Illustrate the hormones and their pathway metabolites that significantly altered among control vs. LY9-treated (e.g., CK vs. 10%, 30%, 50%)

CK vs. 10				
Serial no.	Compound	Fold change	Log_2_FC	Type
1	L-tryptophan	2.16	1.11	up
2	Tryptamine	4.06	2.02	up
3	Indole	2.19	1.13	up
4	2-Methylthio-cis-zeatin riboside	3.58	1.84	up
5	N6-isopentenyladenosine	0.50	− 1.01	down
6	Dihydrozeatin-7-glucoside	4.49	2.17	up
7	Dihydrozeatin-O-glucoside riboside	12.01	3.59	up
8	1-Aminocyclopropanecarboxylic acid	2.83	1.50	up
9	Gibberellin A19	3.50	1.81	up
CK vs. 30				
Serial no.	Compound	Fold change	Log_2_FC	Type
1	L-tryptophan	2.39	1.26	up
2	Tryptamine	6.57	2.72	up
3	Indole	3.01	1.59	up
4	2-Methylthio-cis-zeatin riboside	2.53	1.34	up
5	N6-isopentenyladenine	0.49	− 1.04	down
6	1-Aminocyclopropanecarboxylic acid	3.91	1.97	up
7	Gibberellin A19	6.49	2.70	up
8	L-Phenylalanine	2.02	1.01	up
9	Salicylic acid 2-O-β-glucoside	2.94	1.55	up
CK vs. 50				
Serial no.	Compound	Fold change	Log2FC	Type
1	Tryptamine	8.02	3.00	up
2	Indole	2.63	1.39	up
3	Indole-3-carboxylic acid	0.49	− 1.03	down
4	Indole-3-acetyl-L-aspartic acid	4.04	2.01	up
5	2-Methylthio-cis-zeatin riboside	2.96	1.56	up
6	1-Aminocyclopropanecarboxylic acid	2.81	1.49	up
7	Gibberellin A19	4.85	2.28	up
8	Jasmonic acid	0.39	− 1.38	down
9	Jasmonoyl-L-isoleucine	0.49	− 1.03	down
10	L-Phenylalanine	2.69	1.43	up

### Amino Acid Derived Compounds and Hormone Biosynthesis Pathway Upregulated in Rice

**Fig. 6:**
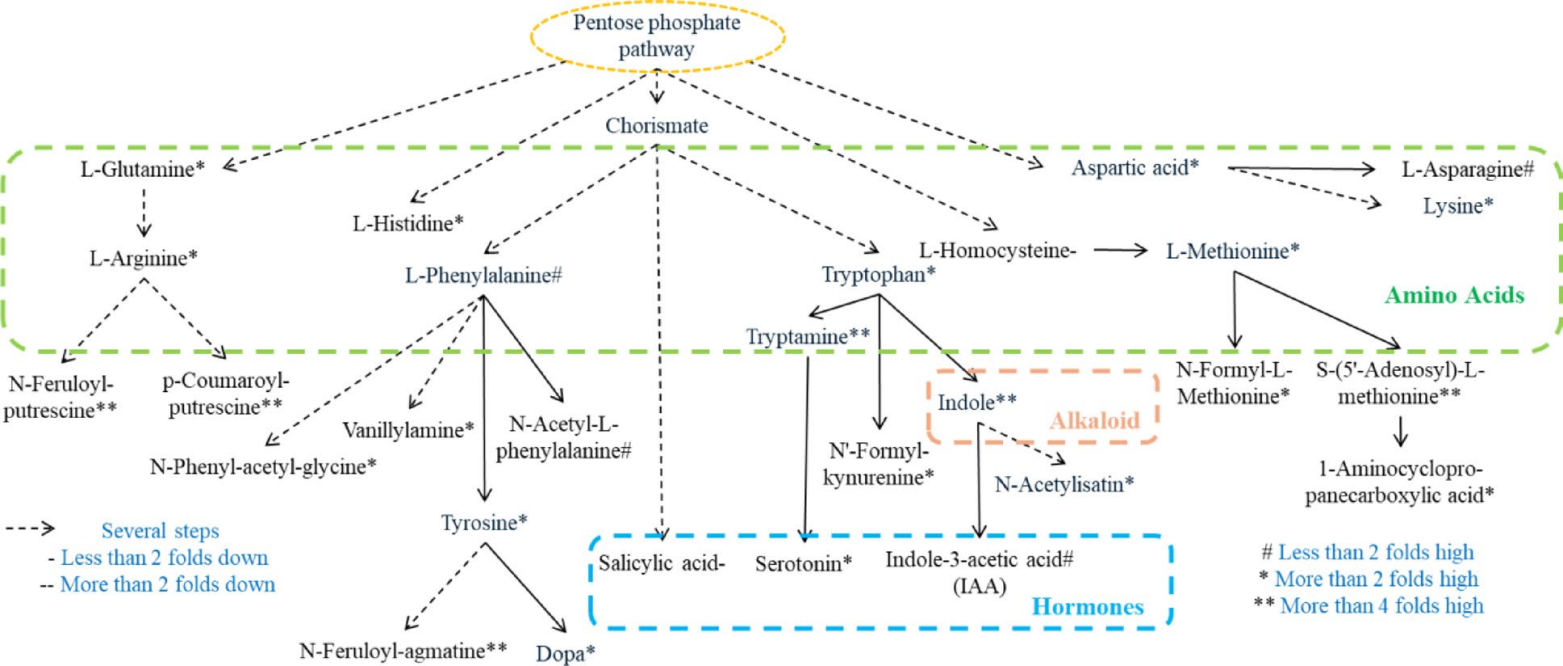
Illustrates the homones and metabolites that altered in rice roots control vs. LY9-treated. The green color represents amino acids and light blue color represents hormones, and different symbols indicate the compounds fold changed in CK vs. LY9

Based on LC-MS/MS metabolomic profiling, we have constructed an intricate metabolic pathway illustrating the biosynthetic networks of amino acids, alkaloids, and phytohormones (Fig. 6). Our analysis revealed that amino acids serve as crucial precursors in the biosynthetic pathways of both alkaloids and hormones, playing a central role in nitrogen metabolism. Notably, we observed a significant upregulation of tryptophan levels, with a more than 2-fold increase in LY9-treated rice roots compared to controls. This elevated tryptophan undergoes a series of enzymatic transformations, first being converted into indole (a nitrogen-containing alkaloid compound), which subsequently serves as an immediate precursor for IAA biosynthesis through the indole-dependent pathway (Fig. 6). The metabolic flux strongly indicates that LY9 treatment induces significant modifications in the expression patterns of nitrogen metabolism-related genes (Fig. 4A, B), leading to enhanced biosynthesis of various nitrogen-containing metabolites (Figs. 5 and 6). The coordinated upregulation of these metabolic pathways results in enhanced root architecture development, increased tillering capacity, and consequently improved overall biomass accumulation in rice plants. These findings provide mechanistic insights into how LY9 treatment modulates primary and secondary metabolism to enhance plant growth and development.

## Discussion

Fairy ring fungi, including *Leucocalocybe mongolica*, *Agaricus arvensis*, *Marasmius oreades*, and *Agaricus campestris*, have garnered significant scientific attention due to their remarkable ability to modify soil properties and substantially influence plant growth and developmental patterns (Fisher 1977; Edwards 1984; Bonanomi et al. 2012; Wang et al. 2022). These fungi facilitate enhanced mineralization of organic materials and promote nutrient accumulation within their fairy ring zones (Bonanomi et al. 2012; Wang et al. 2022). Despite these growth and development-promoting effects are readily observable, however, the underlying physiological and biochemical mechanisms facilitating these alterations within plant remain poorly understood. To address these growth promoting effects in plants, we isolated the LY9 strain () and treated soil with varying concentrations of LY9 to elucidate the underlying mechanisms of plant growth promotion. The results showed that LY9-transformed soil exhibited significant improvements in key physiochemical properties, such as increased total contents of nitrogen, potassium, and available potassium (Fig. 1). Intriguingly, our analysis showed that phosphorus contents and soil pH were decreased in LY9-transformed soil compared to control conditions (Fig. 1). These findings present an interesting contrast to previous research on fairy ring soil associated with *Agaricus* species, which revealed reduced total nitrogen and phosphorus concentrations within fairy ring zones compared to control areas (Caesar-TonThat et al. 2013; Yang et al. 2018). However, the literature presents some contradictory findings, with certain studies reporting elevated available phosphorus levels in fairy rings (Yang et al. 2019). Our observation of decreased phosphorus contents may be attributed to specific environmental conditions and strain-specific characteristics of LY9. Overall, the rice plants cultivated in LY9-treated soil showed enhanced tillering, greener foliage, longer roots, and we identified key genes and metabolic pathways that significantly upregulated in LY9-treated compared to control conditions.

The phenomenon of fairy rings represents a naturally occurring biological system that produces specialized compounds, collectively termed fairy chemicals, which significantly influence plant development. Research showed that these compounds play a crucial role in increasing tillering in rice (Asai et al. 2015) and wheat (Tobina et al. 2014). Our results align with and extend these findings, indicating that rice plants cultivated in LY9-transformed soil exhibited enhanced root development and increased tiller number compared to control plants grown in untreated soil conditions (Fig. 2). This observation is consistent with observed phenotypes of other crops, including rice, wheat, and other grasses, cultivated within fairy ring zones consistently display enhanced developmental characteristics and notably darker green foliage compared to those grown under control conditions (Peter 2006; Duan et al. 2022a). Similarly, our findings provide additional support for these observations, as rice leaves harvested from LY9-transformed soil showed immense dark green color, accompanied by significantly higher levels of chlorophyll ‘a’, chlorophyll ‘b’, total chlorophyll contents, and a higher chlorophyll a/b ratio (Fig. 3).

Previous research has reported that higher expression of genes involved in carbon and nitrogen metabolism pathways serves as a primary driver for activating various metabolic genes that promote rice plant growth (Sun et al. 2013). Our transcriptomic analysis showed significant enrichment of genes associated with amino-acid biosynthesis, nitrogen metabolism, nitrogen-containing compounds, and hormonal signaling transduction pathways in the KEGG pathway enrichment analysis (Fig. 4). Moreover, we found significant upregulation of genes crucial for plant development and metabolism in LY9-transformed rice roots compared to control conditions (Table 1). These upregulated genes involved photosynthesis, chlorophyll pigment synthesis, amino acid metabolism, IAA signaling, alkaloid production, tillering regulation, sucrose transport, light-harvesting chlorophyll a/b-binding (LHCB) protein synthesis, and OsMYB44 transcription factor (Table 1). Recent studies have revealed that upregulation of genes related to nitrogen metabolism, chlorophyll synthesis, and nitrogen-containing compounds significantly improve photosynthetic efficiency and growth characteristics in tomato plants (Sun et al. 2023). A recent study has shown that overexpression of the *OsMYB44* gene enhances UV-B tolerance in rice plants by the activation of tryptamine-associated metabolites (Zhang et al. 2024). In addition, the MYB44 transcription factor has been identified as a key regulator in various plant processes, including sucrose accumulation, flavonoid biosynthesis, root growth promotion, and overall plant development (Wang et al. 2023).

Overexpression of the *OsMYB44* gene significantly modulates the expression profiles of multiple tryptamine metabolism-related genes in rice, including *OsTSα*, *OsTSβ*, *OsTDC1*, *OsTDC3*, *OsTHT1*, and *OsTBT2* (Zhang et al. 2024). This regulatory network results in substantially elevated accumulation of tryptamine amino acid in rice leaf tissues (Zhang et al. 2024). The rice roots harvested from LY9-treated soil showed more than two-fold increase in the concentrations of several amino acids and nitrogen containing compounds including essential amino acids (arginine, tryptophan, methionine, lysine, histidine), aromatic amino acids (L-Dopa, tyrosine), amines (tryptamine), and key signaling molecules (indole, N-acetylisatin, fagaramide, indole-3-acetyl-L-aspartic acid), as well as crucial primary metabolites like glutamine and aspartic acid (Fig. 5A, B). These significantly elevated levels of these amino acids function as essential precursors in the biosynthesis of various secondary metabolites including diverse alkaloid compounds, phytohormones, and various bioactive polyphenols (Häusler et al. 2014; Rao and Zheng 2025; Rao et al. 2025). These metabolites are fundamentally important in orchestrating multiple aspects of plant development, including cellular differentiation, organ formation, and tissue patterning (Baqir et al. 2019). Moreover, they play critical roles in regulating growth patterns through complex hormonal signaling networks and mediating adaptive responses to various environmental stressors through both systemic and localized mechanisms (Baqir et al. 2019; Trovato et al. 2021).

Plant growth regulators play crucial roles in modulating root system architecture and development. Auxin and gibberellin, two essential phytohormones, are particularly significant in orchestrating root growth patterns and meristematic activity (Li et al. 2018; Shtin et al. 2022). These hormones function synergistically to promote primary root elongation, regulate cell division in the root apical meristem, and maintain root gravitropism (Li et al. 2018; Shtin et al. 2022). Auxins are critical for root elongation and lateral root formation, and their elevated precursors suggest that LY9 may stimulate these processes through metabolic reprogramming (Table 2). Our results showed consistent rise in gibberellin A19 further supports this growth-promoting effect (Table 2), as gibberellins are known to synergize with auxins to regulate cell elongation and division (Li et al. 2018; Shtin et al. 2022). The downregulation of cytokinins, which often antagonize auxin action, may further tilt the hormonal balance toward growth promotion. The serotonin signaling pathway has emerged as another key player in root development, specifically in lateral root initiation and emergence. This neurotransmitter-like compound influences various physiological processes, including root system architecture, stress responses, and developmental transitions (Erland et al. 2016). The biphasic response of salicylic acid and abscisic acid indicates a nuanced hormonal adjustment to LY9 treatment. While moderate LY9 concentrations may prime stress-responsive pathways, higher doses could suppress these signals to prioritize growth. The reduction in jasmonic acid and its conjugate, jasmonoyl-L-isoleucine, at 50% LY9 treatment suggests a trade-off between growth and defense responses (Fig. 5C; Table 2). Collectively, these observed metabolic signatures align with the enhanced root development phenotypes and indicate a possible mechanistic link between LY9 treatment and the modulation of hormone-mediated root growth responses. Our comprehensive study elucidated the complex interactions between LY9 and plant growth, providing new insights into the molecular mechanisms underlying these beneficial associations.

## Conclusion

In conclusion, our study determines that LY9-transformed soil significantly enhances rice plant growth and development through complex molecular and metabolic mechanisms. The comprehensive analysis revealed that LY9 treatment improves soil physicochemical properties, particularly organic matter content and nutrient availability, creating optimal conditions for plant growth. The enhanced root architecture, increased tillering capacity, and improved photosynthetic efficiency observed in LY9-treated rice plants were highlighted by significant transcriptional reprogramming of key metabolic pathways. Notably, the upregulation of genes involved in primary metabolism, hormone signaling, and nitrogen metabolism corresponded with elevated levels of amino acids, alkaloids, and phytohormones, collectively linked with enhanced tillering capacity, increased root architecture, and improved photosynthetic efficiency establishing a clear molecular basis for these observed phenotypic improvements. These findings have significant implications for sustainable agriculture and the development of novel biofertilization strategies.

## Electronic Supplementary Material

Supplementary Material 1

Supplementary Material 2

Supplementary Material 3

Supplementary Material 4

## Data Availability

The raw data of RNA-seq are deposited in China National GeneBank (CNGB; https://db.cngb.org/cnsa) under project accession No. CNP0007364.

## Declarations

### Pending Patent Applications

Currently, there are two pending patent applications relevant to the work in this manuscript. The patent application numbers are 202410517649.4 and 202410517671.9, are submitted to the National Intellectual Property Administration of China. These applications are in line with the innovative aspects of our research and reflect our efforts to protect the intellectual property stemming from this study.
