# Supplementary material for: Leucocalocybe mongolica Fungus Enhances Rice Growth by Reshaping Root Metabolism, and Hormone-Associated Pathways
Source: Rice (N Y). 2025 Jun 16;18:52. doi: 10.1186/s12284-025-00813-4 (PMC12170973; doi:10.1186/s12284-025-00813-4)
Supplement: Supplementary file 2 — Supplementary Material 2 [file 12284_2025_813_MOESM2_ESM.docx]

**1.** Preparation of potato dextrose agar (PDA)

1.1 Weighing and cooking

Weigh the culture medium one by one according to the formula, peel the potatoes, cut them into small pieces and put them into a container for later use, add 1000ml of distilled water, Pour into a pot and heat on a heater until boiling, maintain for 20 to 30 minutes. Filter with gauze while hot and discard the filtrate. Filter the residue, take the filtrate and add distilled water to 1000 ml.

1.2 Heating and dissolving

Add 20g of glucose and 15-20g of agar, then place on an asbestos net and heat over low heat, using a glass rod to gently stir. After the agar is completely dissolved, dispense into conical flasks and sterilize at 121 ℃ for 15 minutes.

**2.** LY9 inoculation treatment

In the clean bench, use a sterile inoculation spatula to take a small piece of the existing LY9 strain and culture it on PDA. Select an appropriate position on the substrate surface and fix the mycelium face down on the PDA medium. Well marked and sealed.

**3.** Cultivation

All the culture medium was placed in an incubator at 25°C for 20 days until the surface of the culture medium was covered with hyphae. use.


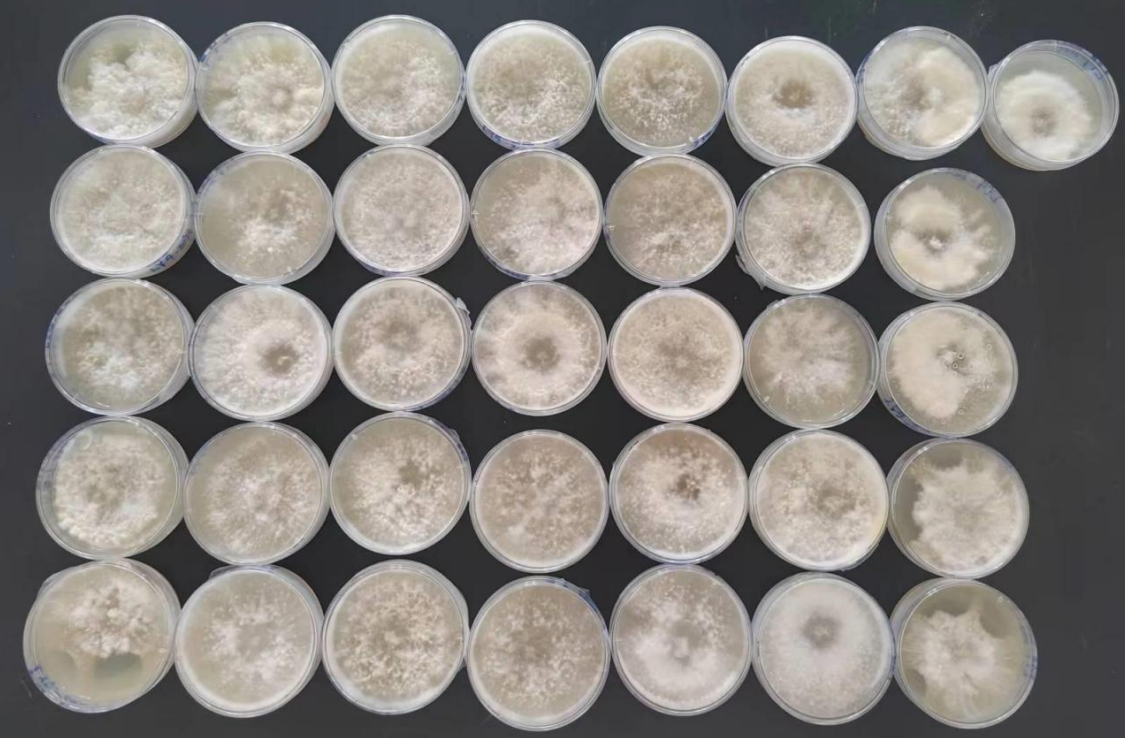


**Supplementary Figure 1**. PDA medium filled with LY9.


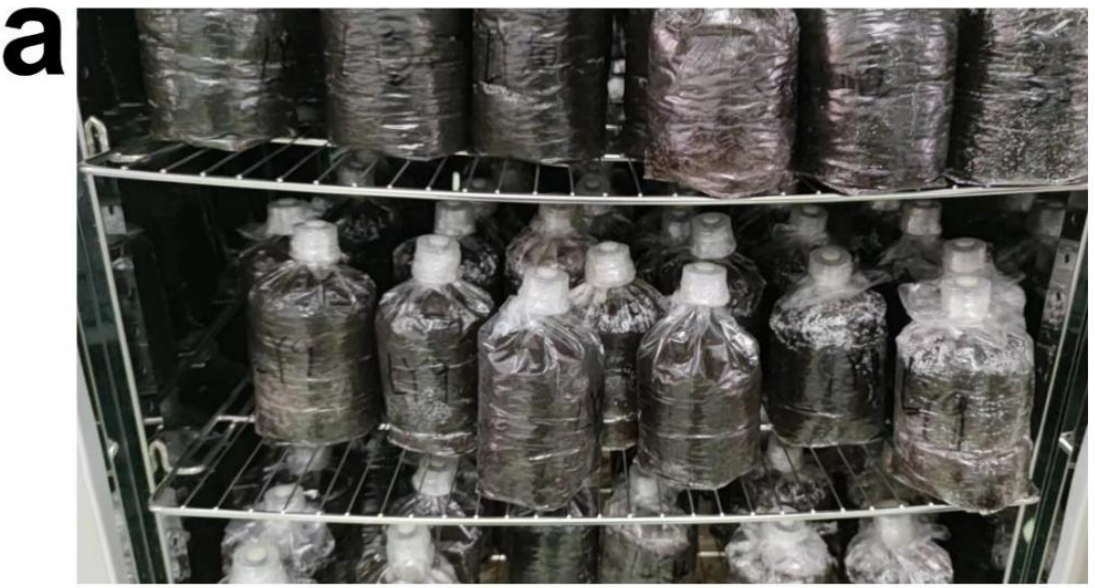

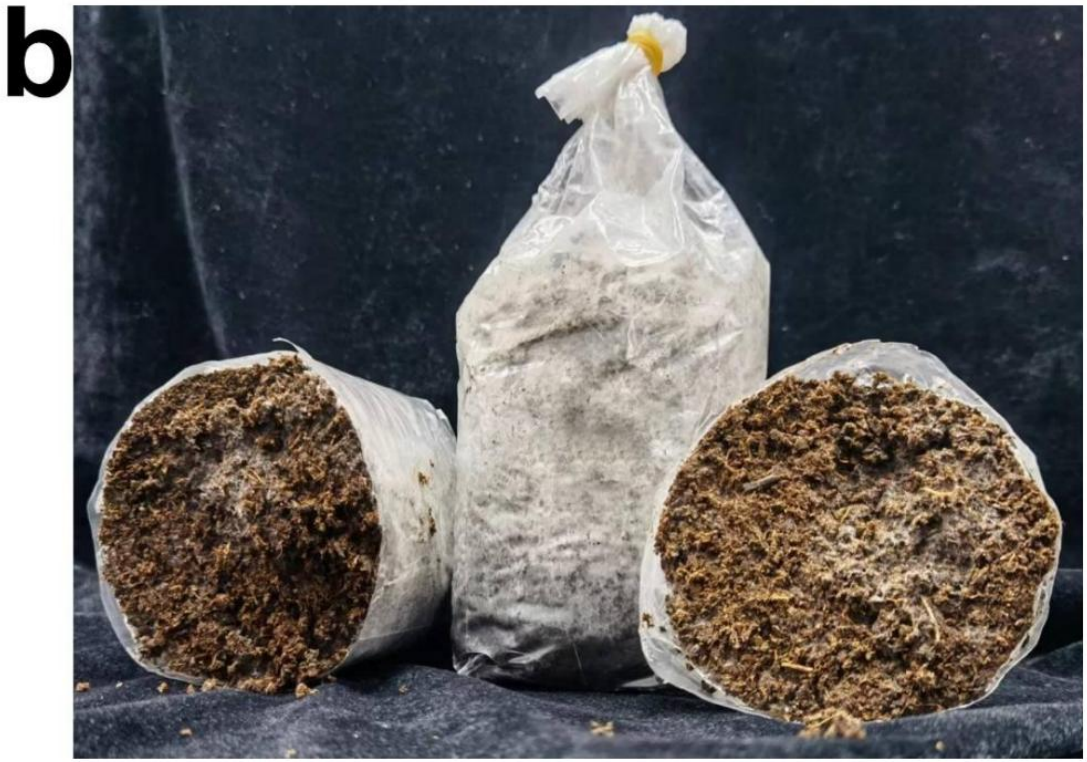


**Supplementary Figure 2.** Preparation of LY9 transformation soil. (a) The process of LY9 transformation soil culture; (b) Surface appearance of LY9 soil after transformation.
